# Outcomes of cemented and hybrid primary total hip arthroplasty for osteoarthritis: A systematic review with narrative synthesis

**DOI:** 10.1007/s00402-025-06007-3

**Published:** 2025-07-28

**Authors:** Amy Pearce, Anna Butcher, Kim Hébert-Losier

**Affiliations:** Te Huataki Waiora School of Health, University of Waikato, Hamilton, New Zealand

**Keywords:** Hip replacement, Quality of life, Survival, Revision, Periprosthetic fracture

## Abstract

**Purpose:**

To compare primary (implant survival and periprosthetic fracture rates, PPF) and secondary (patient reported outcome measures, PROMs) outcomes of cemented and hybrid primary total hip arthroplasty (THA) for osteoarthritis.

**Methods:**

Four databases (PubMed^®^, EBSCO, ScienceDirect^®^, and Scopus^®^) were searched (1 October 2023 and 15 November 2024) for original studies comparing cemented and hybrid primary THA for osteoarthritis. survival, PPF rates, and PROMs. Included studies were assessed for risk of bias using the Quality in Prognostic Studies or RoB 2.0 tool, critically appraised for strength of evidence using GRADE, and underwent a narrative synthesis. PROSPERO registration number CRD42023462884.

**Results:**

Eight studies met criteria for review (*n* = 357,748). Risk of bias was high for two, moderate for three, and low for three studies. Quality of evidence was very low for both primary and secondary outcomes. Five studies met the criteria for the primary outcome (survival) (*n* = 257,756), two PPF rates (*n* = 29,581), and three PROMs (*n* = 382). Three of five studies reported hybrid survival as not significantly different to cemented, and two identified cemented as superior. The three PROMs studies reported no difference between cemented and hybrid THA. A lack of studies and comparative data made it unfeasible to determine PPF outcomes.

**Conclusion:**

Few high-quality studies and methodological heterogeneity led to moderate to high bias and very low overall evidence certainty. Eligible studies indicated no difference in short to medium term PROMs or 10-year survival between the two fixations. Long-term studies indicated superior cemented survival outcomes. A substantial gap in long-term PROMs and PPF rates is noted.

## Introduction

Total hip arthroplasty (THA) is recommended for severe primary osteoarthritis of the hip and is one of the most frequent surgeries worldwide. Wang et al. [1] reported 606.5 million global osteoarthritis cases by 2021, highlighting it as a major public health challenge due to its disability burden. As joint replacement surgeries increase, identifying the most effective and durable treatment approach is essential to ensuring quality of life. THA for osteoarthritis can be performed with cemented (both femoral stem and acetabular cup cemented), cementless (both stem and cup are cementless), hybrid (cemented femoral stem and cementless cup), or reverse hybrid (cementless femoral stem and cemented cup) implants. Controversy over fixation methods for THA has been ongoing for decades, and there seems to be no consensus on which fixation method provides optimal survival and patient reported outcomes (PROMs) despite several systematic reviews and meta-analyses on the topic [2–8]. Most of the reviews in this area exclusively focus on survival and revision rates [9–14], whereas it is recognised that PROMs are an important consideration in establishing THA success and promotion of patient-centred care [15, 16]. In addition, certain of these reviews present methodological concerns where data from the same registry reported in different studies [4], systematic reviews or meta-analyses [4, 6, 8], and registry reports [4] alongside original research were included in the analysis, biasing findings by duplicating data analysed. Making inferences on the superiority of fixation methods for THA outcomes specifically for osteoarthritis is further challenged considering how several of these reviews included additional hip pathologies as criteria for surgery (e.g., lupus, systematic sclerosis, rheumatoid arthritis) [2, 3, 5]; involved hemi-arthroplasty in addition to full THA [3]; limited their review to restricted age groups [5]; or failed to specify whether stems were cemented or uncemented, focusing only on acetabular fixation [2, 7, 8]. We identified four reviews that included PROM comparisons as a secondary outcome to implant survival [2–4, 6], but only one of these exclusively targeted osteoarthritis [4].

The use of cemented THA implants was first described in 1891 and became more common from the 1950 s [17]. Cemented THA has been identified as the most affordable fixation method [2, 4, 18] and is often considered the “gold standard” [4], setting the bar that newer implants need to surpass for widespread adoption. At the other end of the spectrum, cementless THA is considered the most costly fixation method [19] and is typically associated with poorer outcomes [9, 10, 13] and higher revision and periprosthetic fracture rates [20] than cemented. Hybrid THA, despite costing more than cemented, may offer superior postoperative quality of life [21] and function [22]. Consequently, hybrid THA has been identified as the most cost-effective replacement surgery [4] in all age groups and both sexes [18], and hybrids with conventional polyethylene show a survival advantage over cemented THA [23]. Consideration of both surgical and patient-related measures in quantifying success of THA, regardless of fixation method, is needed to shift the focus from surgeon to patient-centred models of care [24].

Given the identified gaps in the existing reviews comparing THA outcomes between fixation methods, this systematic review with narrative synthesis aimed to compare both primary (revision and periprosthetic fracture rates) and secondary (PROMs) outcomes between cemented and hybrid primary THA for severe endpoint osteoarthritis. We hypothesised hybrid THA would demonstrate potentially superior primary and secondary outcomes to cemented THA for patients with end stage osteoarthritis.

## Methods

This systematic review with narrative synthesis forms part of independent research supported by a PhD scholarship award jointly funded by Tauranga Orthopaedic Research and The University of Waikato. This review was designed to meet the 2020 Preferred Reporting Items for Systematic Review and Meta-Analysis (PRISMA) reporting guidelines [25] and was prospectively registered in the International Prospective Register of Systematic Reviews (PROSPERO identifier: CRD42023462884).

Review methods and inclusion criteria were specified in advance. The primary outcomes of interest were survival and/or revision rate and periprosthetic fracture (PPF) rate, and secondary outcomes of interest included any physical criteria PROMs, including but not limited to, the Western Ontario and McMaster Universities Osteoarthritis Index (WOMAC), Oxford hip score, Harris hip score, Forgotten hip score; as well as any mental health or lifestyle related PROMs (HRQOL), such as Hospital Anxiety and Depression Scale, Short Form-36 item health survey (SF-36), and Veterans Rand 12 item health survey (VR-12). Original, peer-reviewed research articles published in English were included, and spanned observational studies (prospective and retrospective) and randomised control trials. We further limited our inclusion criteria to studies evaluating outcomes over a period of at least 1 year. Where multiple studies reported on the same/similar patient cohort, the study with the longest follow-up was included to avoid duplication of data. Study population included adult patients with end stage hip osteoarthritis who had been assigned a fully cemented (cemented femoral stem and acetabular cup) or hybrid (cemented femoral stem and cementless acetabular cup) THA. Revision risk, hazard, mortality, and economic evaluations; editorials; systematic reviews; meta-analyses; joint registry reports; letters to the editor; and conference abstracts were excluded. Studies including any condition other than end stage osteoarthritis of the hip, revision outcomes, hip resurfacing (hemiarthroplasty) or bilateral THA surgeries only were excluded. Studies that included fully cementless (cementless femoral and cementless acetabular cup) or reverse hybrid (cementless femoral stem and cemented acetabular cup) procedures were included when comparisons were made between fully cemented and hybrid THA within the study. A strength of our inclusion criteria ensured that the same dataset was not featured multiple times in different studies thereby inflating results.

Four electronic databases (PubMed^®^, EBSCO, ScienceDirect^®^, and Scopus^®^) were initially searched on 1 October 2023, with the search updated on 15 November 2024. The search included articles from journal inception up to and including the date of search. Broad syntax terms included “primary hip replacement”, “primary hip arthroplasty”, “osteoarthritis”, “cement*”, “hybrid” and “outcome”. Detailed descriptions of the syntax used for each database is provided as supplementary material (Supplementary 1). The database search was complemented by searching reference lists, orthopaedic joint registries, and hip arthroplasty manufacturer websites for relevant articles. All records identified via the database search (AP) were imported to the review software Rayyan [26] and duplicates removed. Two reviewers applied eligibility criteria independently (AP, AB), and then met to discuss conflicts. There was one unresolved conflict that required input from a third reviewer (KHL). The initial screening for eligibility was performed on titles/abstracts, and the process repeated for full-text articles by the same two independent reviewers. A customised data collection form was developed for this review (Supplementary 2) and data collated in Microsoft Excel (v2501, Microsoft Corp., Redmond, WA, USA). Information extracted from each study included study type, fixation method, country of study, sample size, mean follow-up period, primary endpoint, statistical methods for survival calculation, predictors of interest, and results. Missing data were identified, and corresponding authors of these studies were contacted. If there was no response from the authors, data were reported as “missing”. Data were summarised in table format to facilitate narrative synthesis and interpretation (Supplementary 2). Studies were assessed for risk of bias and critically appraised. Two reviewers assessed each study independently (AP, AB), with a third (KHL) available for conflict resolution, but not required. Observational studies were assessed for risk of bias using the Quality in Prognostic Studies (QUIPS) tool [27] and randomised control trials with the Cochrane RoB version 2.0 [28]. The QUIPS is considered a useful and reliable tool for systematically assessing the risk of bias in prognosis research across six key domains (study participation, attrition, factor measurement, confounding, outcome measurement and analysis/reporting). The RoB 2.0 provides a structured framework to evaluate potential bias across five domains relevant to randomised controlled trials (randomisation, deviations from intended intervention, missing outcome, measure of the outcome, selection of the reported result) [28].

Although a meta-analysis was planned to provide a quantitative synthesis of the findings from aggregate data, the decision to perform a narrative synthesis was motivated by six factors: data availability and homogeneity, type of data, study design, purpose of the synthesis, study quality and reporting, and flexibility. As five or more studies using similar statistical methods and reporting the same outcomes from similar populations are needed to achieve reasonable power from a random effect model meta-analysis [29], data extracted as part of this review were deemed insufficient for meta-analysis of primary and secondary outcomes. A narrative synthesis systematically synthesises evidence to generate new insights by analysing and interpreting findings from included studies. The process involves three steps: developing a preliminary synthesis, exploring data relationships, and assessing the robustness of the synthesis [30]. These steps were followed when undertaking our synthesis. The overall quality of evidence for each gradable outcome was assessed by one reviewer (AP) using the Grading of Recommendations, Assessment, Development, and Evaluation (GRADE) working group system [31], and verified by a second (KHL). This structured approach has four levels or certainty of evidence ratings (very low, low, moderate, and high) used to assess risk of bias, imprecision, inconsistency, indirectness, and publication bias. Gradable outcomes were survival/revision rate, PPF rate, and PROMs. Study design, risk of bias assessment, and quality of evidence ratings did not constitute study exclusion. Results were organised based on primary and secondary outcomes. Comparison and synthesis of results for each of these categories of outcome were summarised and categorised as conclusive (either “there is difference” or “there is no difference”) or inconclusive [32] based on heterogeneity, statistical uncertainty, inconsistency in treatment effects, and/or incomplete information.

This review focused specifically on studies reporting survival percentages and revision rates (absolute outcomes), rather than those which modelled the risk of revision over time. We were interested in how many hips had been revised or remained unrevised at a specific timepoint rather than the statistical likelihood of revision. This focus on absolute outcomes allows for clarity and accessibility of easily interpretable data with concrete outcomes [33], relevant for decision making, such as benchmarking (national registry reports use survival percentage at 5, 10 or 15 years to compare models) [11] and clinical planning (surgeons want information on likelihood of a revision, not just the relative risk). This approach also aligns with real-world clinical goals, such as identifying implants that consistently exceed revision benchmarks [11].

## Results

**Fig. 1:**
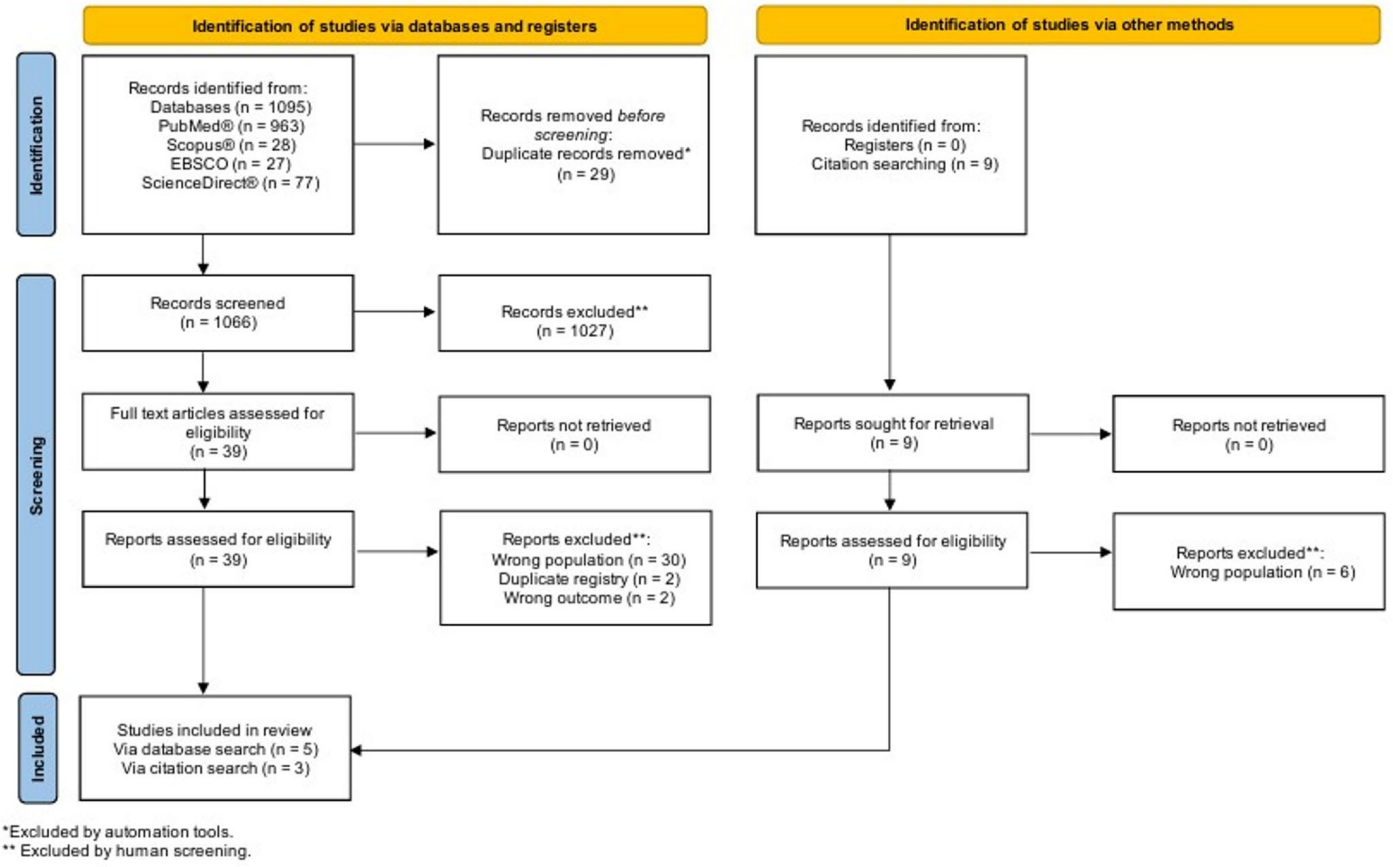
PRISMA flow chart of the search strategy and study selection process

The initial search identified 1095 studies, with 9 studies identified from citation searches. After duplicate removal, 1027 studies were excluded at title and abstract screening (Fig. 1). Ultimately, 8 studies met inclusion. Five of these reported on the primary outcome (all observational), and three these addressed the secondary outcome (two randomised control trials and one observational). We attempted to contact three authors [34–36] for further information, but no replies were received.

### Primary outcome: survival and PPF

#### Risk of bias

The five studies identified were assessed for risk of bias. Direct comparison of sex ratios and mean age were possible in one of five studies, which indicated no difference in proportion of male to female observations (*p* = 0.610) or mean age (*p* = 0.464) [10]. All other studies reported adjusting for age and sex within their statistical models to avoid confounding [9, 11–13]. Three observational studies had independent low risk of bias [10–12], and one indicated moderate risk in prognostic factor measurement and study confounding [13]. One study displayed a high-risk of bias related to confounding and moderate bias in both study attrition and prognostic factor measurement [9]. Detailed results of the individual bias assessments can be found in Table 1.

**Table 1:** Risk of bias assessment

Primary outcome (survival)							Secondary outcomes (PROMs/HRQOL)				
		Bloemheuvel et al. (2022) [9]	Jamsen et al. (2014) [10]	Kandala et al. (2015) [11]	Makela et al. (2008) [12]	Pedersen et al. (2014) [13]	Nilsdotter et al. (2003) [34]			Norman- Taylor et al. (1997) [35]	Onsten et al. (1994) [36]
QUIPS*									ROB 2**		
Study participation		+	+	+	+	+	+		Randomisation process	x	+
Study attrition		!	+	+	+	+	!		Deviations from the intended intervention	+	+
Prognostic factor measurement		!	+	+	+	!	!		Missing outcome data	+	+
Outcome measurement		+	+	+	+	+	!		measure of the outcome	+	!
Study confounding		x	+	+	+	!	!		Selection of the reported result	+	+
Statistical analysis and reporting		+	+	+	+	+	+		Overall	!	!

#### Overall quality of evidence

Due to the heterogenous nature of the observational survival studies, both survival and PFF evidence was determined to be of low quality and certainty. Four studies collected data from participants of different age group categories. Data were collected across multiple timepoints, spanning several years, with studies capturing survival outcomes over 1–5 years, 5–10 years, or 10–16 years, making it difficult determine a summary of primary outcomes. Only age and fixation type consistently appeared as moderating factors. GRADE quality of evidence for the outcome of survival and PPF rate was low, with moderate-to-high risk of bias, serious indirectness, serious imprecision, and suspected publication bias (Table 2).

#### Study characteristics

The five observational studies included in the analysis [9–14] represented 244,032 cemented and 39,068 hybrid THA observations (Table 1). All studies reported on European national joint registry data (four Nordic studies, one England and Wales), and included cemented, hybrid, and cementless survival comparisons, with two studies also examining reverse hybrid THA [9, 13]. There was notable clinical heterogeneity in the age groups studied, with age ranging from 18 to 102 years in the studies that reported age. Two studies focused specifically on patients aged over 80 years [9, 10], one study included individuals aged 55 years and older [12], another targeted patients between 35 and 55 years [13], and one study did not specify the age or age range of participants [11]. Two studies reported overall male and female observations combined for all THA categories within the study [9, 13], while two others provided separate implant observations by sex [10, 12]. One study did not specify or report male and female observations [11]. Study observation periods spanned from 1980 to 2019 with follow-up durations ranging from 1 to 16 years. Survival outcomes were reported at different follow-up periods. Two studies reported survival at 1, 3, and 5 years [9, 10], and one study at 2 years [13]. Four studies reported survival at 10 years [10–13] and one at 16 years [13]. Three studies [9–11] employed a competing-risk Kaplan-Meier approach, while two used standard unadjusted Kaplan-Meier survival analysis [12, 13], reflecting methodological heterogeneity. Two studies reported PPF rates [9, 10]. Detailed characteristics can be found in Table 3.

**Table 2:** Certainty of evidence GRADE* for primary and secondary outcomes

Certainty assessment							Summary of findings			
Outcome	Studies (*n*)	Study design	Risk of bias	Indirectness	Imprecision	Publication bias	Included in narrative synthesis		Results	Quality of evidence (GRADE)
Survival	5	Observational studiesª	moderate-high	serious^f^	serious^ij^	suspected^l^	Hybrid: 39,068 Cemented: 204,964		No difference in survival rates heterogeneity in population/samples	+ - - -
PROMs	3	2 Randomised Control trials^b c^; 1 Observational study^d^	moderate- high	serious^fgh^	serious^i^	suspected^m^	Hybrid: 138 Cemented: 247		No significant difference between hybrid and cemented PROMs. Old studies, poorly conducted with limited results.	+ - - -
PPF	2	Observational studies^e^	moderate-high	serious^fe^	serious^k^	suspected^n^	Hybrid: 3,745 Cemented: 25,836		Lowest in hybrid compared to cemented. Extremely limited study number	+ - - -

*GRADE- grading of recommendation, assessment, Development and evaluation; RCT- randomised controlled trial.

^a^ 3/5 studies did not justify sample size, study attrition, prognostic factor measurement and study confounding; ^b^ One study did not identify the randomisation process and deviated from the intended intervention; ^c^ Studies did not effectively measure the intended outcome; ^d^ The only observational study did not clearly define study attrition, prognostic factor measurement, statistical analysis and reporting used a mean follow up; ^e^ Study designs were not specific to periprosthetic fracture rate, this was an additional outcome; ^f^ Differences in age categories of all studies; ^g^ Two studies used the same PROMs instrument but reported the results differently; ^h^ Different HRQOL instruments; ^i^ Time point collection for each study was different; ^j^ Studies had differing predictors of interest and varied between short, medium and long term net and cumulative statistical survival calculation; ^k^ Periprosthetic fracture rate only recorded if part of revision, not recorded if revision not required; ^l^ National registries represented, results may not be attributable to other populations; ^m^ Only two small samples represented in the RCTs, from the same country; ^n^ Only reported in 2/8 studies.

**Table 3:** Survival study characteristics between cemented and hybrid primary total hip arthroplasty for osteoarthritis

Author and year	Study period and country	Study design	Sample size* (cemented; hybrid)	Age group (years)	Mean follow up, collection time points (years), collection end	Outcomes	Predictors of interest	Key findings
Bloemheuwel et al. 2022 [9]†	2007–2019 Netherlands¹	Observational. Cemented, hybrid, cementless, reverse hybrid	*n* = 43,053 (22,025; 3,243)	≥ 80	Mean: 4.2 (0–13) Times: 1, 3, 5 End: Revision	Competing risk Kaplan Meier; Revision rate (%)	Age, sex, ASA, fixation, head size, approach	No difference in survival at either 1, 3 or 5 years. PPF rates cemented 6%, and hybrid 3%.
Jämsen et al. 2014 [10]†	1998–2009 Finland²	Observational. Cemented, hybrid, cementless.	*n* = 4,777 (3,811; 502)	≥ 80	Mean: 4 (1–13) Times: 1, 3, 5, 10 End: Revision	Competing risk Kaplan Meier % survival.	Age, sex, facility, year, comorbidity type, popularity of implant	No difference in survival at 1, 3, 5 or 10 years. PPF 11% in cemented, 18% cemented women vs. 0% hybrid.
Kandala et al. 2015 [11]	2003–2012 England and Wales³	Observational. 2 cemented, 2 cementless and 1 hybrid.	*n* = 239,089 (137,990; 28,471)	Not reported	Mean: not reported Times: 1, 5, 10 End: Revision	Competing risk Kaplan Meier; Bathtub, Weibull, Loglogistic, Flexible Revision rate (%) ‡	Age, sex, fixation, bearing type	Conclusive. Reported new 5% benchmark for revision. Revision rate of 1.88–2.11% in cemented and 2.42% in hybrid.
Makela et al. 2008 [12]	1980–2004 Finland²	Observational. 2 cementless, 1 cemented and 1 hybrid.	*n* = 50,968 (34,296; 3,784)	55–64, 65–74, > 75	Mean: 4.5–9.2 Times: 10, 15, 20 End: Revision	Kaplan Meier standard % Survival	Implant groups, age, sex	No difference. All age groups in both hybrid and cemented groups had a survival of > 90% at 10 years.
Pedersen et al. 2014 [13]	1995–2011 Sweden, Denmark, Finland, Norway⁴	Observational. Cemented, cementless, hybrid and reverse hybrid.	*n* = 29,558 (6,842; 3,068)	35–45 & 46–55	Mean: not reported Times: 2, 10, 16 End: Revision	Unadjusted Kaplan Meier % survival at 2, 10 and 16 years	Age, sex, fixation technique and calendar year	Conclusive. Cemented indicated higher survival percentage at 2, 10 and 16 years for any revision reason, revision for aseptic loosening and revision for any other cause.

ASA- American anesthesiologist physical status rating; PPF-Periprosthetic fracture rate.

All studies included cementless/uncemented and/or reverse hybrid fixation or more than one cemented/hybrid component for comparison.

*Total sample size includes all hip fixation methods within the study, including cementless and reverse hybrid, where applicable.

** Specified a loaded-taper or composite beam stem.

† These studies included periprosthetic fracture rates.

‡ Actual revision rates at 5 years and estimated 10-year rates for 70-year-old males and females only.

¶ No data at 15 or 20 years for hybrid fixation. The fixation technique did not fulfil the proportional hazards assumption for Cox proportional hazards calculation; therefore, the population was split into subgroups (1995–1999, 2000–2003, 2004–2007, 2008–2011).

¹ Dutch arthroplasty register; ² Finnish Arthroplasty Registry; ³ National registry for England and Wales; ⁴ Nordic arthroplasty registry.

#### Narrative synthesis

Three survival studies with the lowest risk of bias [10–12] identified no significant difference in survival between cemented and hybrid THA at 1, 3, 5, and 10 years. All three studies presented mean survival for each fixation method, which ranged between 98.6 and 98.8% at 1 year, 98.1 to 98.3% at 3 years, 97.8 to 98.1% at 5 years, and 83 to 96% at 10 years. Due to heterogenous methodologies, these survival percentages were not directly comparable. In an England and Wales 10-year follow up study [11], after completing five different Kaplan-Meier model analyses, the cemented prostheses with metal-on-polyethylene bearing surface had the highest estimated mean survival rate of 98.02% at 10 years, with cemented ceramic-on-polyethylene survival second with 97.5%, and hybrid metal-on-polyethylene third with 97.32%. With similar 10-year estimates between prostheses and a revision percentage below 5%, the authors recommended the 10-year benchmark revision rate for all THA in England and Wales be lowered from 10 to 5% [11]. No difference in survival between hybrid and cemented THA was reported, although not assessed for statistical significance. Makela [12] identified that at 10 years, all age groups in both hybrid and cemented THAs exceeded a survival of 90% with no significant difference between the two fixation categories across age groups (*p* = 0.22 to 0.80). A moderate risk of bias study [13] reported survival comparison at 10 and 16 years for both cemented and hybrid THA using three endpoints: revision for any reason, revision for aseptic loosening, and revision for other causes. Cemented THA was reported to show superior survival to hybrid THA at 10 years when the endpoint was revision for any reason (90.2 ± 0.43% versus 86.6% ± 0.69), for aseptic loosening (92.8% ± 0.38 versus 91.4% ± 0.59) and for other reasons other than aseptic loosening (97.3% ± 0.23 versus 94.8% ± 0.44). The same pattern was reported at 16 years, with cemented exhibiting higher survival than hybrid. Statistical significance results were not reported. Synthesis of results seems to indicate that at timepoints up to 10 years, survival tends to be similar and that there is little, if any, difference in survival between cemented and hybrid implants, but that longer time frames may favour cemented THA. Two studies reported PPF rates [9, 10], with one of these studies only reporting cemented PPF rate and not the hybrid PPF rate. The low risk of bias study [10] reported a 10-year cemented PPF revision rate of 11%. The high risk of bias study [9] reported a PPF rate of 6% in cemented and 3% in hybrid THA at 5 years [10], thus it is not possible to definitively establish whether PPF rates are lower in cemented or hybrid THA. Both studies showed no difference in survival between hybrid and cemented THA [9, 10]. Detailed survival study information can be found in Table 4.

**Table 4:** Survival and revision rate study outcomes

Study (Age group)	Sample size	Periprosthetic fracture rates	1-y survival %	2-y survival %	3-y survival %	5-y survival %	10-y survival %	15-y survival %	16-y survival %
Bloemheuwel et al. 2022 [9]^ab^	**Cemented**: 22,025	26/427 (6%)	Net:98.7 (98.6–98.8)^gh^	-	Net: 98.2 (98.1–98.3)^gh^	Net: 97.8 (97.7–97.9)^gh^	-	-	-
≥ 80 years			CR: 98.7 (98.6–98.9)^fh^		CR: 98.3 [98.1–98.4]^fh^	CR: 98.0 (97.7–98.2)^fh^			
Median age: 83 (80–108)^e^									
M:10,931; F: 32,073^e^							-	-	-
			Net: 98.6 (98.4–98.8)^gh^	-	Net: 98.2 (98.0-98.4)^gh^	Net: 98.0 (97.7–98.3)gh			
Median FU: 4.2y	**Hybrid**: 3,243	2/61(3%)	CR: 98.8 (98.3–99.1)^fh^		CR: 98.3 [97.8–98.7]^fh^	CR: 98.1 [97.5–98.6]^fh^			
Jämsen et al. 2014 [10]^ab^	**Cemented**: 3,811	5/46 (11%) 1y	CR:98.8 (98.4–99.2)^fh^	-	CR: 98.1 (97.7–98.6)^fh^	CR: 97.8 (97.3–98.3)^fh^	CR: 97.4 (96.9–98)^fh^	-	-
≥80y	M: 1,143; F: 2,668	10/57(18%) in F							
	Mean age: 82y (80–102)								
	**Hybrid**: 502	0/5(0%) in F	CR: 98.6 (97.6–99.7)^fh^	-	CR: 98.1 (96.9–99.4)^fh^	CR: 98.1 (96.9–99.4)^fh^	CR: 98.1 (96.9–99.4)^fh^	-	-
Mean FU: 4y	M: 145; F: 357								
	Mean age: 82y (80–95)								
Kandala et al. 2015 [11]^abcd^	**Cemented**: 137,990	-	-	-	-	-	CeCoP: 98.12^f^ (97.89–98.03)^j^	-	-
Age not specified	M: -; F: -						CeMoP: 97.78^f^ (97.17–97.58)^j^		
Median age: 70.4y^e^									
	**Hybrid**: 28,471	-	-	-	-	-	HyMoP: 97.58^f^ (96.92–97.50)^j^	-	-
Mean FU: -	M: -; F: -								
							**Aseptic loosening** ^**j**^	**Aseptic loosening** ^**j**^	
							55-64y: 85 (84–86) *n* = 2,138	55-64y: 71(69–73) *n* = 1,086	
							65-74y: 91 (91–92) *n* = 5,899	65-74y: 85(84–86) *n* = 2,049	-
							≥ 75y: 95 (95–96) *n* = 2,299	≥ 75y 93(92–94) *n* = 441	
							All: 91 (91–91) *n* = 10,335	All: 83(82–84) *n* = 3,575	
	**Cemented**: 34,296						**All reasons** ^**j**^	**All reasons** ^**j**^	
Makela et al. 2008 [12]^ad^	M: 12,004; F: 22 292	-	-	-	-	-	55-64y: 83 (82–85) *n* = 2,140	55–64: 68(66–70) *n* = 1,088	
	Mean age: 70y (55–95)						65-74y: 89 (89–90) *n* = 5,904	65–74: 82(81–83) *n* = 2,051	-
							≥ 75y: 93 (93–94) *n* = 2,300	≥ 75y: 91(90–92) *n* = 441	
55- >75y							All: 89 (89–89) *n* = 10,343	All: 80(80–81) *n* = 3,579	
							**Aseptic loosening** ^**j**^		
	**Hybrid**: 3784	-	-	-	-	-	55-64y: 90 (85–95) *n* = 77		
	M: 1,551; F: 1,548						65-74y: 93 (91–95) *n* = 194	-	-
mean FU: 3.6-10.2y	Mean age: 72y (55–96)						≥ 75y: 96 (93–100) *n* = 73		
							All: 93 (92–95) *n* = 343		
							**All reasons** ^**j**^		
							55-64y: 83 (76–89) *n* = 78		
							65-74y: 88 (86–91) *n* = 194	-	-
							≥ 75y: 92 (88–95) *n* = 73		
							All: 88 (86–90) *n* = 344		
	**Cemented**: 6,842	-	-	Any: 98.6 ± 0.14^i^	-	-	All: 90.2 ± 0.43^i^	-	All: 77.4 ± 1.13^i^
Pedersen et al. 2014 [13]^a^				AL: 99.6 ± 0.43^i^			AL: 92.8 ± 0.38^i^		AL: 80.5 ± 1.13^i^
35-55y				Other: 99.0 ± 0.12^i^			Other: 97.3 ± 0.23^i^		Other: 96.1 ± 0.39^i^
Mean age: -									
M: 15,391^e^				Any: 97.7 ± 0.27^i^			All: 86.6 ± 0.69^i^		All: 68.5 ± 2.12^i^
F: 14,167^e^		-	-	AL: 99.3 ± 0.15^i^	-	-	AL: 91.4 ± 0.59^i^	-	AL:75.9 ± 2.17^i^
Mean FU: -	**Hybrid**: 3,068			Other: 98.4 ± 0.23^i^			Other: 94.8 ± 0.44^i^		Other: 90.1 ± 1.08^i^

AL- Aseptic loosening; CeCoP- Cemented ceramic on polyethylene; CeMoP- Cemented metal on polyethylene; CR- Competing risk (death); F- Female; FU- Follow up; M- Male.

^a^ Included other hip types (cementless and/or reverse hybrid) in the comparison; ^b^ Only included specific makes of component; ^c^ Included bearing surface as a predictor of survival; ^d^ Bilateral THR not mentioned; ^e^ Of the entire sample only; ^f^ Crude survival rate - Competing risk survival rate with death as a competing risk; ^g^ Net survival rate- The estimated survival rate accounting for only those who survived long enough to require revision potentially; ^h^ Mean survival% [95%CI][low - high]; ^i^ Mean survival% ± standard deviation; ^j^ Range of survival percentages between Bathtub, Weibull, Loglogistic and flexible Kaplan-Meier models.

### Secondary outcome: patient reported outcome measures

#### Risk of bias

Three studies [34–36] were identified as including PROMs and evaluated for risk of bias. The only observational study [34] was evaluated using the QUIPS tool [27], and the two remaining RCTs [35, 36] evaluated using the ROB 2 [28]. Baseline comparisons showed no age or sex differences between cemented and hybrid groups in the Swedish RCT [36] (low risk of confounding). In the UK RCT [35], the hybrid group was significantly younger than the cemented group (*p* < 0.001, d = 0.97), indicating possible age-related confounding. The Swedish observational study [34] had more females and older participants in the cemented group, suggesting imbalance in key demographic variables. While both RCTs showed comparable sex ratios (*p* > 0.05) [35, 36], the observational study did not [34]. These differences lower confidence in the observational findings due to risk of confounding and contribute to downgrading the certainty of evidence. The observational study [34] indicated a moderate risk of bias in study attrition, prognostic factor and outcome measurement, and study confounding, with all other factors indicating a low risk of bias. Both RCTs scored moderate risk of bias overall [35, 36]. Individual high-risk of bias was identified in the randomisation process in one of the RCTs where randomisation was not described [35]. Results can be found in Table 1.

#### Overall quality of evidence

The overall certainty of evidence was very low based on GRADE for secondary outcomes (PROMs) when comparing hybrid and cemented THA, with moderate-to-high risk of bias, serious indirectness, serious imprecision, and suspected publication bias (Table 2). All three studies indicated methodological heterogeneity, using assorted and inconsistently applied tools at varying follow-up points (1, 2, and 3.6 years), and across heterogenous populations with poorly defined or non-existent subgroups. These factors severely limited the validity, comparability, and applicability of findings to clinical practice.

#### Study characteristics

Characteristics of individual studies can be found in Table 5. The three studies [34–36] together compared PROMs scores between 138 hybrid and 247 cemented primary THAs (186 males and 200 females) for osteoarthritis. Two RCTs (*n* = 80 and *n* = 60 respectively) [35, 36], and one observational study (*n* = 196) [34] were performed in Europe (two in Sweden [34, 36] and one in the UK [35]) and compared specific hybrid and cemented implants rather than broad category components. Observation periods ranged from 1988 to 1998 and reported short-term (1, 2, and 3.6-year) PROMs, with two studies also reporting preoperative scores [34, 35]. Both RCTs [35, 36] used the Harris hip score measurement tool, however one RCT converted the original Harris score to Rosser Index pain and disability values [35], and reported Rosser Index quality of life results rather than Harris hip score.

**Table 5:** Characteristics of included studies comparing proms in cemented and hybrid total hip arthroplasty for osteoarthritis

Author	Study period and country	Study design	Sample size	Age group	Outcomes	Follow-up period	Collection time points	Predictors of interest	Key findings
Nilsdotter et al. (2003) [34]	1995–1998 Sweden	Observational†	*n* = 196 C-139 H- 57	NR	HRQOL: SF-36 PROM: WOMAC PreOp: Yes	3.6 y	1 and 3.6y	Age, sex, fixation, follow up time, baseline values	No difference in PROMs between cemented and hybrid THR noted. Statistically significant improvement in postoperative scores for both fixation types.
Norman- Taylor and Villar (1997) [35]	1992–1994 United Kingdom	RCT	*n* = 80 C- 40 H- 40	< 70	HRQOL: Rosser Index PROM: Harris Hip Score PreOp: Yes	1 y	1 y	Age, sex	Inconclusive. Harris hip score used to determine Rosser index score
Onsten et al. (1994) [36]	1988–1991 Sweden	RCT	*n* = 60 C- 30 H − 30	40–70 y	HRQOL: No PROM: Harris Hip Score PreOp: No	2 y	2 y	Fixation	No difference in PROMS between cemented and hybrid THA noted.

C- Cemented; H- Hybrid; THA- Total hip arthroplasty; NR- Not reported; PROMs- Patient reported outcome measures; RCT- Randomised Control Trial; SF-36- 36 Item Short Form Survey; WOMAC- Western Ontario and McMaster Universities Arthritis Index.

† Hospital sample

The results of the Swedish RCT study [36] indicated a 2-year mean Harris pain score of 42 out of 44 for each THA group, and mean Harris hip score of 91 and 92 for hybrid and cemented respectively, with no reporting of statistical significance. In the UK RCT [35], there was no statistically significant difference between the cemented and hybrid preoperative Rosser Index HRQOL scores (*p* = 0.596); but at 1-year, hybrid THAs performed statistically significantly better than cemented THAs (0.988 ± 0.018, 0.995 ± 0.006 respectively, *p* = 0.015) with the cemented hip reporting better Rosser Index HRQOL scores. The study determined a hybrid prosthesis did not impair early quality of life outcomes and although HRQOL scores increased in all 80 patients, the study identified that 60% of hybrid THA patients achieved maximal HRQOL scores after 1 year. The study, however, suggested improvement in quality of life was not dependent on prosthesis type.

The observational study [34] included preoperative, 1-year, and 3.6-year WOMAC and SF-36 scores. No difference in WOMAC scores between the cemented or hybrid groups at any timepoint was noted. The SF-36 subscales of “role physical” and “general health” showed statistically significant differences (*p* < 0.05) preoperatively, and in all but one subscale (i.e., “bodily pain”) at 1-year, and two subscales (i.e., “bodily pain” and “general health”) at 3.6 years with hybrids indicating better scores. However, when the scores were adjusted for age, sex, follow-up time and baseline values, no statistically significant differences were revealed. See Table 6 for detailed results of each study.

#### Narrative synthesis

With very low certainty of evidence, in three heterogenous studies using assorted PROMs tools at inconsistent timepoints, drawing secondary outcome conclusions regarding superiority is challenging. All three studies report PROM improvements in both cemented and hybrid THAs at their chosen follow-up periods, and no significant differences in PROMs between the two THA options. Despite differences in instruments and follow-up durations, the consistent direction of effect (i.e., no observed or statistically significant difference) supports the conclusion that PROMs may be similar between the two fixation types. While formal data synthesis was not feasible, our narrative synthesis suggests that across different instruments and timepoints, PROMs do not appear to differ meaningfully between cemented and hybrid THA. However, due to high risk of bias and very low certainty of evidence, these finding must be interpreted with caution. The lack of observable difference may be due to study limitations and imprecision.

**Table 6:** Outcomes. Of PROM studies

Article	Prosthesis type	PROM (mean ± SD)							HRQOL(mean ± SD)			
		Instrument	Sub level	PreOp	1 year	2 years	3.6 years	Instrument	Sub level	PreOp	1 year	3.6 years
									PF	30.43 ± 20.4	61.6 ± 22.4	56.5 ± 24.2
									RP	6.8 ± 17.1	51.7 ± 42.3	39.7 ± 42.9
									BP	30.5 ± 15.6	72.3 ± 24.9	64.9 ± 25.6
			Pain	45.3 ± 17.9	84.4 ± 17.1	-	81.0 ± 20.1		GH	66.4 ± 19.6	68.9 ± 20.5	64.4 ± 21.1
	Cemented (*n* = 177)		Stiffness	37.7 ± 16.6	75.8 ± 20.5	-	76.1 ± 22.0		VT	47.8 ± 21.3	68.7 ± 22.0	61.0 ± 24.0
	Mean age: 75 (61–92)¶		Function	37.1 ± 15.5	75.4 ± 18.0	-	71.3 ± 20.4		SF	62.6 ± 26.5	84.7 ± 23.5	81.0 ± 23.0
	M: 73; F: 104								RE	33.6 ± 41.0	63.3 ± 40.7	56.8 ± 44.0
									MH	68.3 ± 19.8	80.2 ± 19.3	76.5 ± 19.8
Nilsdotter et al. (2003) [34]		WOMAC						SF-36				
*n* = 245									PF	30.4 ± 17.7	74.2 ± 19.7*	68.2 ± 25.3*
Age: NR	Hybrid (*n* = 68)								RP	15.5 ± 28.7*	72.4 ± 37.1*	63.9 ± 41.6*
	Mean age: 62 (50–72)¶								BP	30.3 ± 20.0	78.0 ± 21.7	69.2 ± 27.9
	M: 39; F: 29		Pain	44.8 ± 15.9	84.1 ± 18.1	-	84.2 ± 21.0		GH	72.4 ± 20.3*	78.7 ± 21.6*	70.1 ± 23.9
			Stiffness	42.0 ± 15.7	81.8 ± 16.6	-	80.7 ± 22.3		VT	51.2 ± 20.2	78.0 ± 20.8*	70.0 ± 24.1*
			Function	41.8 ± 12.5	83.0 ± 16.1	-	81.2 ± 20.4*		SF	67.0 ± 25.3	92.7 ± 17.2*	90.6 ± 20.5*
									RE	43.8 ± 44.3	86.3 ± 29.2*	83.6 ± 32.0*
									MH	71.6 ± 23.8	87.3 ± 15.7*	83.4 ± 20.1*
										A B C D	A B C D	
									I	-	15, 3, 3,	-
									II	8, 3	4, 10, 2, 1	-
	Cemented (*n* = 40)								III	1, 8	‘ 1, 1	-
	Mean age: 58.22y ± 6.7 (40–65)		Pain	-	-	-	-		IV	3, 17	-	-
	M: 19, F: 21		Hip	-	-	-	-		V	-	-	-
Norman-Taylor & Villar (1997) [35]		Harris Hip†						Rosser Index	VI	-	-	-
*n* = 80										A B C D	A B C D	-
Age: <70y	Hybrid (*n* = 40)		Pain	-	-	-	-		I	-	24, 6	-
	Mean age: 52.90y ± 6.6 (39–67)		Hip	-	-	-	-		II	5, 10	5, 3, 1	-
	M: 22, F: 18								III	2, 8	1	-
									IV	2, 13	-	-
									V	-	-	-
									VI	-	-	-
	Cemented (*n* = 30)		Pain	-	-	42	-					
	M: 16; F: 14		Hip	-	-	91	-					
	Mean age: 63 ± 6‡											
Onsten et al. (1994) [36]		Harris Hip‡		-	-		-	-	-	-	-	-
*n* = 60	Hybrid (*n* = 30)		Pain	-	-	42	-					
Age: 40-70y	M: 17; F: 13		Hip	-	-	93	-					
	Mean age: 62 ± 6‡											

*P < 0.05 between hybrid and cemented mean outcome score.

BP-Bodily pain; GH-General health; HRQOL- Health related quality of life; MH- Mental health; PROM- Patient reported outcome measure; PF-Physical function; RE- Role emotional; RP- Role physical; SD- Standard deviation; SF- Social function; SF-36-36 Item Short Form Survey; VT- Vitality; WOMAC-Western Ontario and McMaster Universities Arthritis Index.

† Actual scores not reported, used to determine Rosser index.

‡ No range provided.

¶ No standard deviation provided.

¶ The Rosser index derives scores from the Harris hip Score, the matrix allocates scores from − 1.486 to 1.000. A score of 1 indicates complete normality; death is given a score of 0.000. Numbers reported are the distribution of patients within each category with AI being the best score and DVI being the worst quality of life score.

## Discussion

This review focused on studies comparing implant survival, PPF rate, and PROMs between hybrid and cemented primary THA for end stage osteoarthritis. From the eight retrieved studies [9–13, 34–36], five observational studies [9–13] compared the primary outcomes of survival and two studies compared PPF rate [9, 10]. Two RCTs [35, 36] and one observational study [34] analysed PROMs.

### Survival

While the overall number of observations in the survival studies was substantial (*n* = 283,100), lack of clinical and methodological homogeneity led to variability in findings. Three studies [9, 10, 12] found no survival difference between hybrid and cemented THA over 1, 3, and 5 years, while two [11, 13] reported marginally better 10-year cemented survival and one study [13] better 16-year cemented survival. However, neither of the latter studies confirmed whether the observed differences reached statistical significance. Survival outcomes appeared to vary by patient group, follow-up duration, and implant type. Studies examined patients from under 55 years only [13] to those over 80 years only [9, 10]. In some studies, survival seemed to be better in specific cemented subgroups (e.g., ceramic-on-polyethylene bearings) [11], suggesting that individual implant characteristics may be more important than fixation type alone.

Broad category classifications of THA fixation groups include multiple implant brands (stems and acetabular cups), designs (geometry), materials, and coatings, which can behave differently in terms of longevity, wear, and complications [37]. Examining broad categories can highlight general trends, but lacks the detail needed to guide implant selection. Research on specific implant comparison may allow more accurate assessment of variability in patient outcomes, improve implant selection for patients, provide more clinically relevant outcomes, and avoid misleading conclusions [38]. Focusing on specific implant comparisons risks reduced sample sizes and statistical power, compromising inference, but the continued use of regional or national registry data could mitigate this limitation by leveraging access to larger and more generalisable patient populations.

Only two studies reported PPF rates [9, 10], both in patients 80 years and older but at different timepoints. One study indicated higher PPF rates in cemented THA [9]. Still, this difference did not translate into poorer implant survival in cemented THA overall. The available data do not support PPFs as the most common cause of revision in either cemented or hybrid THA. More importantly, clinical failures that did not result in revision were not documented. Since some PPFs are managed without revision (e.g., with fixation), and these cases were not reported, the true incidence of PPFs may be underestimated in both fixation types. This lack of reporting lowers the proportion outcome of PPF in cemented and hybrid fixation in THA. A study analysing data from the UK National Joint Registry found that 56% of postoperative PPFs were not captured by the registry, as the cases were treated without the replacement of components [39]. Lamb et al. [39] noted PPF as the leading cause of major reoperation (but not necessarily component replacement) following THA and that the prevention and treatment of PPF requires further research. It may be prudent to include, at a minimum, PPFs requiring surgical intervention within revision registry data collection.

All survival studies [9–13] used national registry derived data for survival analysis. The lack of consistent reporting of statistical significance, follow-up periods, and patient stratification reduces the overall ability to determine meaningful inferences from findings. While national joint registries are invaluable for monitoring and improving the outcomes of joint replacement surgeries, they do have some limitations. National registries provide broad standardised data that help identify overall trends in outcomes, implant longevity, and patient demographics [40, 41]. However, they mask regional disparities by averaging data across different populations and may not capture variations, for example, between wealthier and deprived regions or populations of different ethnicities. Regional joint registries may offer more detailed insights into regional population characteristics, specific access barriers, rural versus urban areas, and variations in surgical and funding outcomes (private versus public) [42]. For surgeons, regional data analysis may be more effective at determining the most successful THA for a specific demographic of patient.

Methodological heterogeneity between the survival studies complicated comparison and inference. Standardising survival analysis models, the handling of missing data, statistical adjustments, and definition of outcomes affected the validity and comparison of results. Methodological homogeneity in registry data analysis would allow opportunity for easier comparison and evaluation of survival outcomes.

### Patient reported outcome measures

Although limited, all PROMs studies [34–36] reported improvements in HRQOL following primary THA for osteoarthritis, regardless of fixation type. One RCT [35] found a statistically significant advantage for hybrid THA at 1-year using the HRQOL Rosser Index. The observational study [34] reported differences in SF-36 subscales, favouring hybrid THA at 1-year and 3.6-years, but these differences were not statistically significant after adjusting for age, sex, follow-up time, and baseline values. Overall, both hybrid and cemented PROMs improved substantially and appeared similar between groups, but due to a small number of studies and no follow-up beyond 3.6 years, no difference in PROMs between hybrid and cemented THA could be identified in the medium-term to long-term. The included studies spanned a 30-year period and dated timeframe (1988–1998) and offer valuable historical perspectives. However, their relevance to contemporary practice is limited due to outdated measurement tools, evolving surgical techniques, and shifting patient expectations. Interpretation should be cautious, particularly when comparing to modern cohorts. In all three studies [34–36] included in our systematic review, younger, likely more active patients, were selected for hybrid THA, possibly reflecting surgeon preference rather than a truly random comparison. This bias creates indirectness of evidence as outcomes may reflect patient characteristics rather than implant design.

The absence of comparable PROM data of more than 3.6 years also highlights a major evidence gap. A seven-year prospective international multicentre study observed that while patients experienced significant improvements in PROMs within the first-year post THA, certain factors like obesity and postoperative difficulties in self-care were associated with a gradual decline in outcomes over the subsequent years [43]. This study highlights the importance of continued monitoring to assess the sustainability of surgical benefits. Research also indicates that PROMs tend to plateau after 1-to-2 years post-surgery, but that attrition over time may affect study sensitivity [44]. This attrition rate could be mitigated by using prospective national or regional population PROMs data rather than smaller sample groups [45]. A longitudinal cohort study by Schwartz et al. [46] emphasised that cognitive and mental health assessment significantly influence recovery trajectories post THA and suggested that long-term PROMs are essential for identifying patients at risk of poorer outcomes and for tailoring postoperative care accordingly. Such information is vital for setting realistic patient expectations and facilitating informed decision making regarding the procedures available. Although one RCT reported statistically significant HRQOL differences at 1 year favouring hybrid THA [35], this finding was not consistently observed across other studies or timepoints.

There is a pressing need for contemporary, high-quality research evaluating patient-centred outcomes across different fixation types using standardised PROMs. Future studies should standardise the selection, collection and reporting of PROMs ideally in alignment with internationally agreed guidelines. While the Oxford hip score is one of the most used hip-specific PROM tool for THA [47], valued for its simplicity, validation and strong correlation with patient satisfaction and outcomes, the WOMAC is considered the highest quality PROMs tool for osteoarthritis [48]. The authors propose the use of at least one of these tools as an international standard. Transparency on missing data, loss to follow-up, and selection bias is essential. Lack of studies could reflect a systematic under collecting and underreporting of PROMs in this area contributing to uncertainty and limiting evidence-based decision making. Our recommendations would be to collect PROMs preoperatively, at 6 months to 1-year, and then at 5 yearly intervals, in line with the New Zealand Joint Registry [49].

Although RCTs are the gold standard for evaluating the efficacy and safety of medical interventions, Pakvis et al. [7] argued that RCT studies are challenging to conduct in THA and may not always reflect the complexities of real-world orthopaedic care. These authors suggested that well-designed non-randomised studies with adequate follow-up and rigorous outcome measurement could serve as a benchmark for high-quality evidence in orthopaedics [7]. These benchmark studies would involve comparing similar populations, interventions and follow-up durations, using consistent outcome tools, data collection timelines, methodologies, and standardised statistical analyses.

Both survival and PROMs studies suffered from common limitations, including clinical and methodological homogeneity and inconsistent reporting. There are key limitations in the current literature, notably few published studies; heterogeneity in study design, outcome measures, and follow up durations; and all studies conducted in European countries. The fact that all studies in our review were European, limits the international generalizability of the findings due to potential differences in healthcare systems, surgical techniques, patient demographics, and ethnic variations in hip anatomy [50,51]. Broadening the evidence base to include studies from regions such as North America, Canada, Africa, Asia and Oceania would promote more equitable and optimal patient outcomes across diverse global populations.

Bias assessments identified moderate-to-high and high risk of bias across studies, with concerns related to confounding, prognostic factor measurement, randomisation and missing outcome data. Additionally, publication bias is suspected due to the low number of eligible studies. Taken together, the evidence comparing hybrid and cemented THA is limited by study quality, short follow-up in PROM studies, and inconsistent methodology in survival studies. While both fixation types appear to offer good early PROMs and comparable implant longevity, further research is required– particularly longer term PROMs studies and implant survival at the prosthesis level [52] rather than broad fixation categories. Standardised methods, adjusted analyses, and stratification by patient and implant characteristics are essential for informed decision making.

## Conclusion

Our review of the literature identified primary survival and PPF rates and secondary PROMs outcomes between hybrid and cemented THA for patients with end stage osteoarthritis from eight available studies. The studies do not clearly demonstrate a superior fixation category between hybrid and cemented THA. Both THA options showed improvements in early PROMs, exceeding 98% survival at 1 and 2 years and at least 90% survival at 10-years. All studies identified were European and evaluated outcomes from 1980 to 2019, suggesting that earlier studies might not relate to current surgical practices. Indeed, changes in practice over time may influence the heterogeneity of this review. The included studies were also inconsistent in reporting similar patient demographics, particularly age, PROMs, study time periods, length of follow up, statistical methods, and THA implant components. European only studies are limited in their ability to reflect outcomes in non-European settings, due to regional variations in clinical practice and patient demographics. The absence of longer-term PROM data is a significant gap and likely the biproduct of short-term focus, resource, and time constraints. Future research should focus on product specific outcomes, standardized methodologies, and longer PROMs follow up to provide clearer insights. Regional joint registry data can enhance decision making by considering demographic and socioeconomic factors for more personalised treatment procedures. This review is the first to attempt to summarise literature contrasting survival, PPF, and PROM outcomes between cemented and hybrid primary THA for osteoarthritis, identifying comparable outcomes between methods when considering the few studies available.

## Data Availability

No datasets were generated or analysed during the current study.
